# Comprehensive molecular profiling of intrahepatic cholangiocarcinoma in the Chinese population and therapeutic experience

**DOI:** 10.1186/s12967-020-02437-2

**Published:** 2020-07-06

**Authors:** Longrong Wang, Hongxu Zhu, Yiming Zhao, Qi Pan, Anrong Mao, Weiping Zhu, Ning Zhang, Zhenhai Lin, Jiamin Zhou, Yilin Wang, Yongfa Zhang, Miao Wang, Yun Feng, Xigan He, Weiqi Xu, Lu Wang

**Affiliations:** 1 Liver Surgery Department, Shanghai Cancer Center, Fudan University, No. 270 Dongan Rd., Shanghai, 200032 People’s Republic of China; 2 Shanghai Cancer Center, Fudan University, Shanghai, People’s Republic of China

**Keywords:** Intrahepatic cholangiocarcinoma, Next-generation sequencing, Molecular profiling, Target therapy, Immune therapy

## Abstract

**Background:**

The genomic alterations of intrahepatic cholangiocarcinoma (ICC) in the Chinese population have not been fully revealed. Molecular profiling may provide a reference for clinical management, especially targeted therapy.

**Methods:**

A retrospective study was conducted in 122 ICC patients. All patients’ samples underwent next-generation sequencing (NGS), which analyzed 417 genes. The genetic characteristics, clinical management and therapeutic responses were analyzed.

**Results:**

The most commonly mutated genes were TP53 (34%), KRAS (25%) and ARID1A (17%). Targeted agents were used referring to molecular profiling, in combination with chemotherapy. Twenty-two patients with wild-type KRAS/NRAS/BRAF were treated with cetuximab. The disease control and response rates were 78% and 47%, respectively, which were higher than those achieved with chemotherapy alone (72% and 11%, P = 0.16). Fifty-four patients underwent anti-VEGF treatment with bevacizumab. The disease control and response rates were 85% and 60%, respectively. Better therapeutic efficiency (P = 0.001) and longer progression-free survival (PFS) were observed in the bevacizumab-treated group compared to chemotherapy alone group (15.4 and 6.7 months, respectively; P = 0.04). The PFS of ten patients who underwent hepatectomy after combined treatment with chemotherapy and bevacizumab was longer than that of 139 patients who underwent surgical treatment (28.9 vs 18.0 months, P = 0.03). Two patients (1.6%) had signatures of microsatellite instability (MSI-H), and both benefited from immunotherapy.

**Conclusions:**

This study provides an overview of genetic alterations in Chinese ICC patients and indicates the potential clinical implications for NGS-based personalized therapies.

## Background

The incidence of liver cancer ranks 4th among the Chinese population according to data published by the National Central Cancer Registry of China (NCCR) [1]. Intrahepatic cholangiocarcinoma (ICC) is the second most common type and accounts for 10–20% of all primary liver cancers [2, 3]. The increasing incidence rate and aggressive clinical course of ICC contribute to its high mortality [4]. Surgical resection remains the mainstay of potentially curative treatment for patients with early-stage disease, but few treatment options are available for the majority of patients with advanced-stage or unresectable disease. The combination of gemcitabine and cisplatin is still the standard of care for patients with advanced and metastatic disease, and no standard targeted therapy has been proven in clinical trials [5–7]. Pembrolizumab and pemigatinib have been currently approved to treat 10% of patients with specific genetic characteristics, while precise treatments are urgently needed to improve the survival of the remaining 90% of patients with advanced disease.

Next-generation sequencing (NGS) is an ideal tool to categorize patients with ICC based on molecular profiles [8], and few large-scale sequencing studies have focused on the genomic characteristics of ICC in the Chinese population. The molecular phenotypes of ICC have not yet been revealed and represent a rational personalized therapeutic approach.

In this study, NGS was performed on 122 Chinese ICC patient samples to elucidate the molecular profiles, and target or immune agents were administered based on the genetic characteristics.

## Methods

### Patients

Patients were identified over a 4-year period starting in April 2015 and were deemed eligible for the study if they had a confirmed histological diagnosis of ICC. Written informed consent for tumor profiling was obtained from each patient upon their first admission to Fudan University Shanghai Cancer Center (FUSCC). The study protocol was approved by the FUSCC ethics committee (No. 218-1611 and No. 050432-4-1911D).

The clinical data and NGS results for 122 patients with ICC were available at the time of analysis. Overall survival (OS) and progression-free survival (PFS) rates were collected.

Survival data of 139 patients accepted curative surgery for ICC in the same center was used.

### Sample collection and preparation

Previously collected fresh tissue and blood samples were used in this study. The tissues were obtained through laparoscopic surgery or core needle biopsy. The fresh tissue was soaked in 5 times the volume of 4% formaldehyde solution within 30 min. A wax block was made within 24 h after soaking the tissue, and it was sent to the pathologist for diagnosis and review. The specimens were sent to the laboratory for NGS detection within 48 h at 4–8 °C. Twenty milliliters of peripheral blood was drawn and sent to the laboratory within 48 h at 15–35 °C.

Tissue samples with an estimated tumor purity < 10% based on histopathological assessment were deemed insufficient for sequencing. The standard amount of DNA input was 250 ng, and the minimum input was 50 ng in cases for which the DNA quality was limited. Matched germline DNA from prospectively collected blood samples was analyzed for all patients.

### Tissue and plasma DNA isolation and purification

Genomic DNA (gDNA) was extracted from formalin-fixed, paraffin-embedded (FFPE) samples using the GeneRead DNA FFPE Kit (Qiagen, USA), and gDNA was extracted from the white blood cell samples using the DNA Blood Midi/Mini kit (Qiagen, USA). The quality of purified DNA was assayed by gel electrophoresis and quantified by the Qubit^®^ 4.0 fluorometer (Life Technologies, USA).

### Library construction and bioinformatics analysis

Purified gDNA was first fragmented into DNA pieces approximately 200–300 bp in size using an enzymatic method (5X WGS Fragmentation Mix, Qiagen, USA). After end repair, tailing and T-adaptor ligation by polymerase chain reaction (PCR) was used to generate a prelibrary, and the products were then subjected to exon capture. Captured fragments were subsequently purified and hybridized by a 417-gene panel (Additional file 1: Table S1). FASTP [9] was used to trim adapters and remove low-quality sequences to obtain clean reads, which were aligned to the Ensemble GRCh37/hg19 reference genome by BWA [10]. PCR duplicates were processed by GenCore [11], and consensus reads were generated. SAMtools [12] was utilized for the detection of single-nucleotide variations (SNVs), insertions and deletions, and Human Genome Variation Society (HGVS) variant descriptions were annotated by ANNOVAR [13] software. After annotation, SNVs with a PopFreqMax > 0.05 were excluded, and nonsynonymous SNVs with a variant allele frequency (VAF) > 0.5% or a VAF > 0.1% in cancer hotspots collected from the patient database were retained for further analysis.

The microsatellite instability (MSI) statuses of all tissue samples were determined, and this score was used to classify the samples into three groups, MSI-high, ≥ 2 unstable microsatellite loci; MSI-low, only 1 instable locus; and microsatellite stable (MSS), no locus instability. The MSI-high results were further confirmed by PCR validation.

The tumor mutational burden (TMB) was estimated by somatic nonsynonymous mutations per megabase of the panel sequences examined.

Pathway enrichment was conducted in KEGG website (https://www.kegg.jp).

### Immunohistochemical analysis of PD-L1

The tissue was placed in a 4% paraformaldehyde solution for 12 h. Then, the tissues were dehydrated with 75% and 95% absolute ethanol each for 1.5 h at 60–70  °C. Next, the tissues were soaked in 40  °C dichloromethane for 4 h and then rehydrated with absolute ethanol, 95% ethanol, 75% ethanol and distilled water for 1 h, 0.5 h, 1 h and 1 h, respectively, at 60–70  °C. The samples were then embedded in paraffin wax, and ultrathin Sects. (5 µm) were cut using an ultramicrotome (Lecia RM2126RT, Germany), mounted on glass slides, and stained with hematoxylin and eosin for analyzing tissue structures using an upright fluorescence microscope (Eclipse TE2000-S, Nikon, Japan).

We performed immunohistochemical studies to evaluate programmed death ligand 1 (PD-L1) expression on tumor cells (TCs) and immune cells (ICs) using the Ventana SP263 assay with the Ventana BenchMark GX system (Roche/Ventana Medical Systems, Tucson, USA) according to the recommended protocol. A rabbit monoclonal anti-human PD-L1 antibody (clone SP263, Roche/Ventana) was used. The slides were immersed in acetone (3 min) and xylene (10 min) to remove the coverslip; the sections were then rehydrated with alcohol in decreasing concentrations and immersed in distilled water. Antigen retrieval was performed with Cell Conditioner 1 for 64 min against SP263. The sections were then incubated with the specific primary antibody for 16 min against SP263. Subsequently, the sections were treated with the OptiView HQ Linker for 8 min and the OptiView HRP Multimer for 8 min. Finally, counterstaining was performed with Mayer’s hematoxylin and Scott’s tap water bluing reagent. The evaluation of the stained tissue sections was performed by two investigators who had no knowledge of the patients’ clinical status. Cases with discrepancies were jointly re-evaluated until a consensus was reached. PD-L1 expression was calculated as the percentage of membrane staining on TCs or ICs in the overall area of the tumor, regardless of intensity.

### Clinical management

Gemcitabine-based chemotherapy was used as the first-line chemotherapeutic treatment in this study, and combinatory strategies included cisplatin, oxaliplatin and capecitabine. Some patients accepted additional target/immune therapies (detailed in Additional file 2: Table S2). Patients refused the suggested target/immune therapies would accept chemotherapy alone. The Common Terminology Criteria for Adverse Events (CTCAE) 4.0 criteria were used to evaluate adverse events.

Follow-up was conducted every 8 weeks at the lowest frequency. Enhanced abdominal CT/MR scans and serum CA19-9 levels were examined, and Response Evaluation Criteria in Solid Tumors (RECIST) 1.1 criteria were used to evaluate therapeutic efficacy.

### Statistics analysis

To evaluate the association of clinical characteristics or genes, Fisher’s exact test was performed. Odds ratios and false discovery rate (FDR)-corrected P values were also calculated. PFS was calculated using the Kaplan–Meier method, and the Chi square test was used to compare therapeutic efficiencies between patients treated with different strategies and between those with different genetic alterations. The PFS rate was calculated as the time from the treatment start date to the date of progression or death. For patients who underwent surgical procedures, recurrence after curative resection or progression after palliative surgery was considered progression. Patients alive and without progression were censored to the date of the last follow-up.

## Results

Samples from 122 individual patients with ICC were analyzed. Clinical characters are summarized in Table 1.

**Table 1:** Clinical characteristics (n = 122 patients)

Clinical Characteristics	Number (%)
Sex	
Male	72 (59)
Female	50 (41)
Age	
Median (range)	61 (33–89)
Metastasis	
Lymph node	61 (50)
Intrahepatic	46 (38)
Lung	30 (25)
Abdomen/peritoneum	26 (21)
Bone	23 (19)
Invasion	
Diaphragm	16 (13)
Gallbladder	6 (5)
Adrenal gland	4 (3)
Colon	2 (2)
Duodenum	2 (2)
Stomach	1 (1)
Differentiation	
Poor	20 (16)
Moderate	80 (66)
High	22 (18)
Biopsy	
Laparoscopic	31 (25)
Percutaneous	91 (75)
Sample analyzed	
Tissue	111 (91)
Blood	11(9)

**Fig. 1:**
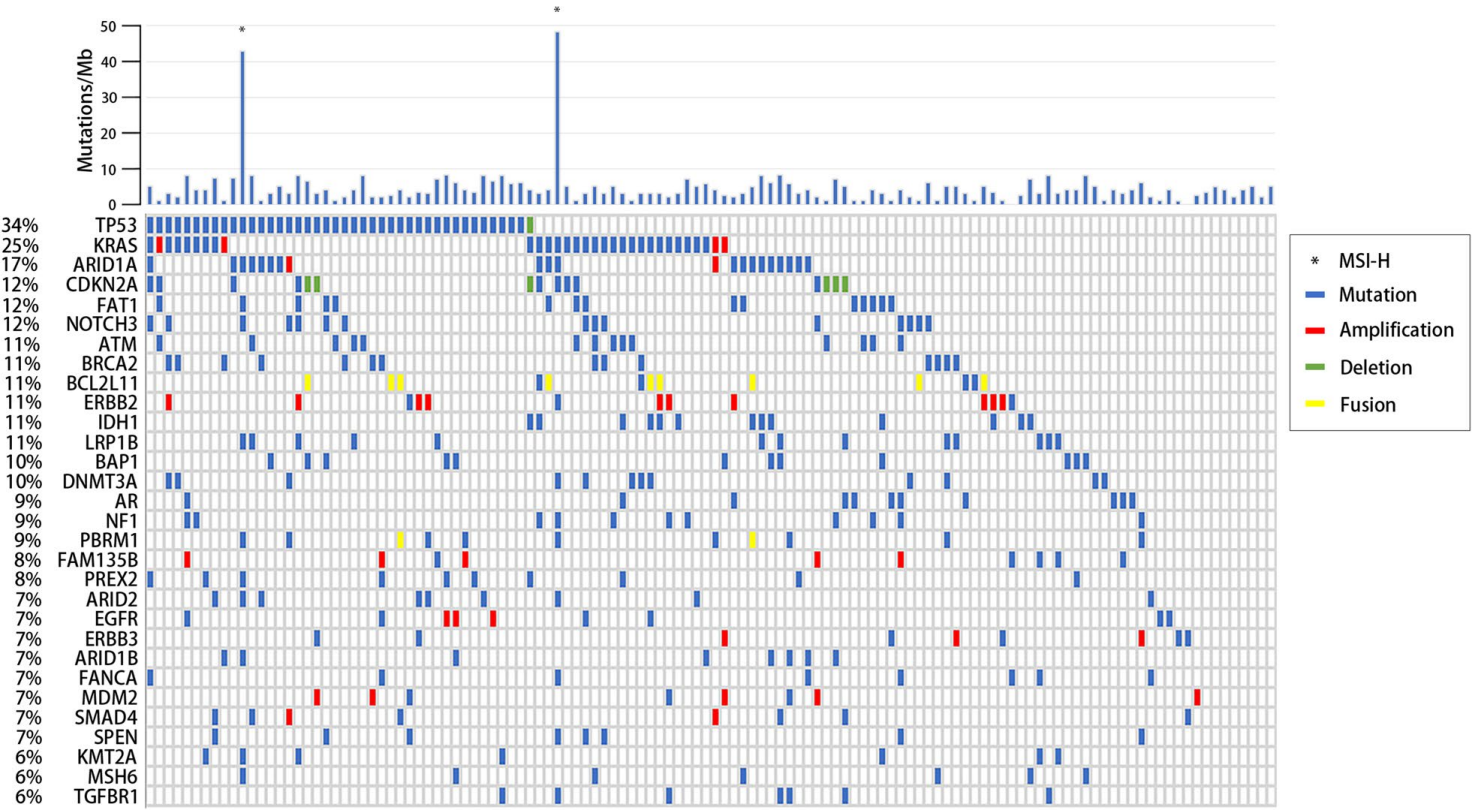
Common alterations and tumor mutational burden

**Fig. 2:**
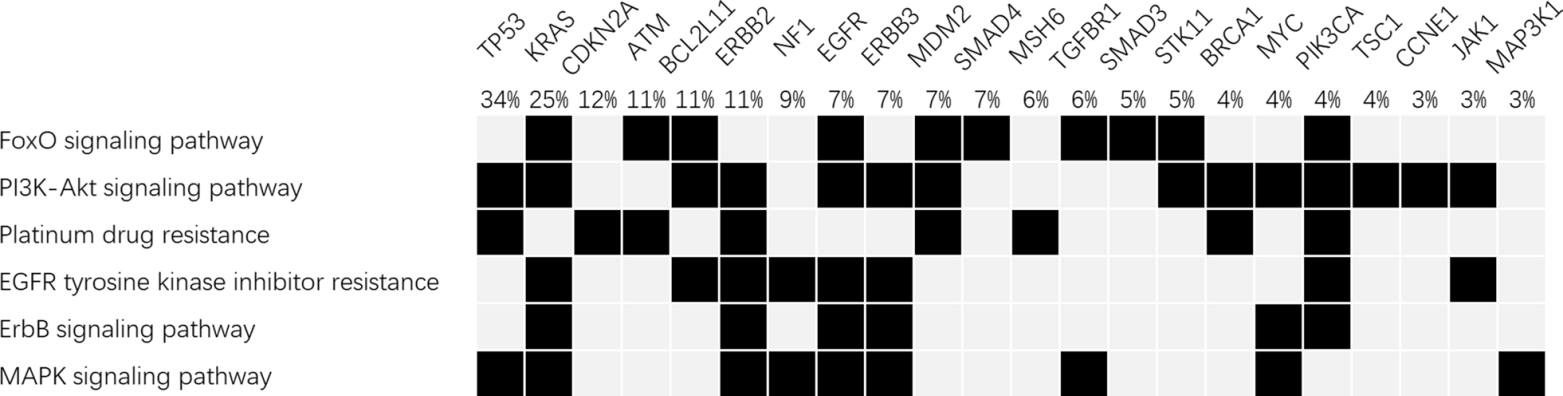
Enriched pathways

A total of 692 genetic alterations were identified among 121 of the 122 samples, while no somatic genetic alterations were identified in the remaining sample. The median number of mutations per sample was 5, and the most commonly mutated genes were TP53 (34%), KRAS (25%), and ARID1A (17%). Among multiple genes, potentially oncogenic focal copy number variations were noted, including ADAM29 (5%) and CDKN2A (5%) deletions and ERBB2 (8%), CDK12 (4%), FAM135B (4%), FRS2 (4%), and MDM2 (4%) amplifications. Rearrangements were mostly noted in BCL2L11 (7%, Fig. 1 and Table 2). Potentially actionable alterations were enriched in FoxO signaling pathway, PI3K-Akt signaling pathway, platinum drug resistance, EGFR tyrosine kinase inhibitor resistance, ErbB signaling pathway, and MAPK signaling pathway (Fig. 2).

**Table 2:** Commonly amplified/deleted/rearranged genes

Gene	Number (%)
Amplification	
ERBB2	10 (8)
CDK12/FAM135B/FRS2/MDM2	5 (4)
CCNE1/KRAS	4 (3)
Deletion	
ADAM29/CDKN2A	6 (5)
NTRK1/NTRK3	2 (2)
NRG3/TP53/MLH1/SMARCA2	1 (1)
Rearrangement	
BCL2L11	9 (7)
PBRM1	2 (2)
ALK/FGFR3(-TACC3)	1 (1)

**Fig. 3:**
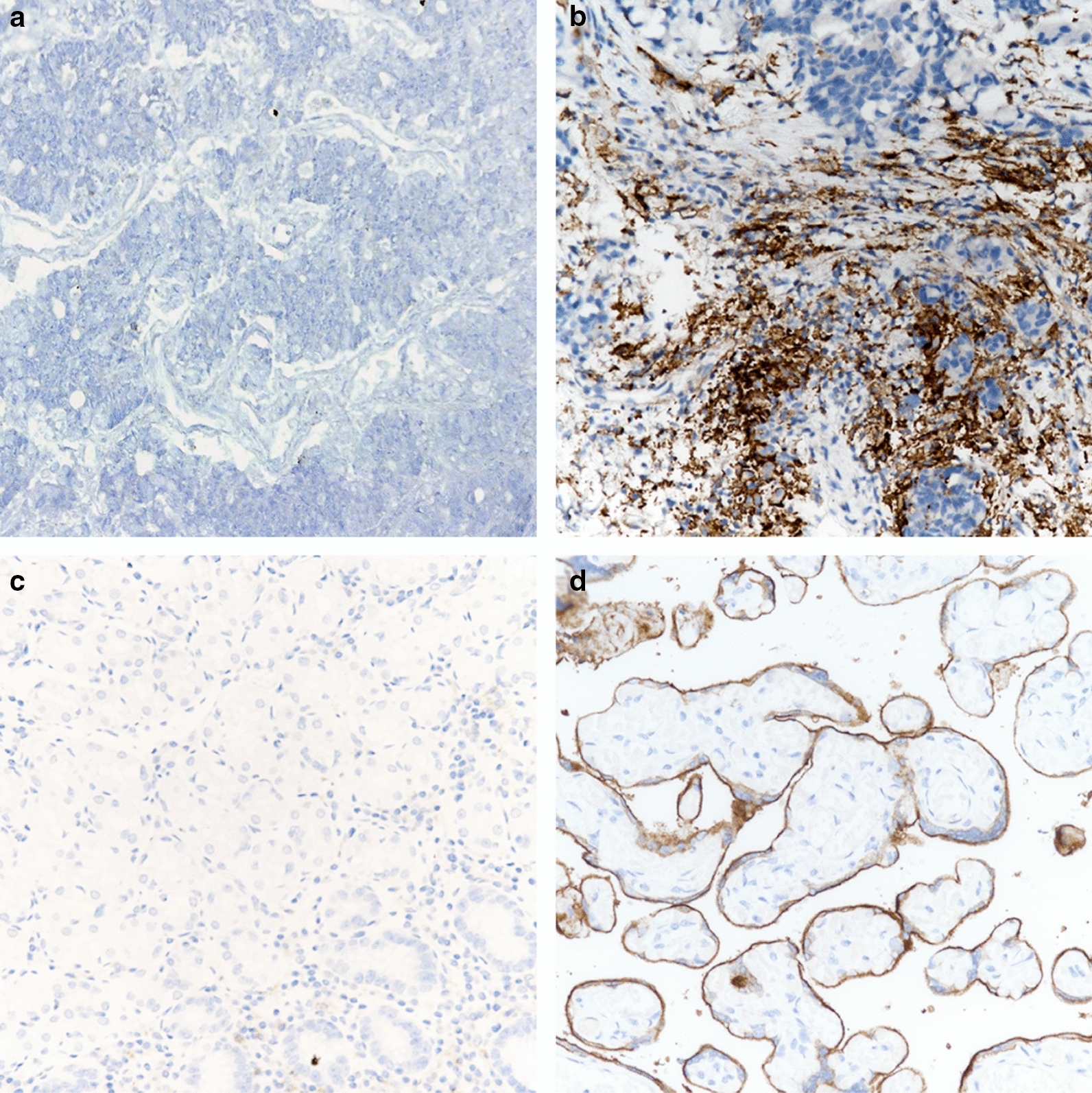
The immunohistochemical results for PD-L1 were negative in patient sample A (**a**) and positive (1%) in patient sample B (**b**). Negative control (**c**) and positive control (**d**)

Two patient samples (1.6%) had MSI-H signatures, and the immunohistochemical results for PD-L1 were negative in patient sample A and positive (1%) in patient sample B (Fig. 3). The total immunohistologically-positive PD-L1 rate in tumor tissues was 16%, and the median expression rate in a single tumor was 5% (range 1–25%). The TMBs for patients A and B were 42 and 48 mutations/Mb, respectively. Patient A was a 57-year-old male with a history of hepatitis B virus (HBV) infection, and he was diagnosed with local advanced left lobe cholangiocarcinoma with intrahepatic metastasis, vessel invasion and lymph node metastasis. The patient was treated with pembrolizumab for 8 months, and he accepted stereotactic body radiation therapy after the 8th treatment cycle. During radiation therapy, the patient was diagnosed with transient hyponatremia and accepted supportive treatment. The patient exhibited a partial response (Additional file 3: Figure S1) and was alive as of the analysis date. Patient B was a 46-year-old male with a history of HBV infection, and he was diagnosed with local advanced left lobe massive cholangiocarcinoma and lymph node metastasis. After 4 cycles of pembrolizumab treatment, the tumor was deemed stable and shrank by 7%. A left hemihepatectomy without lymphadenectomy was performed at 4 weeks after the 4th treatment cycle, and the 5th treatment cycle began at 4 weeks after the surgery. The patient was alive without tumor metastasis as of the analysis date.

ERBB2 alterations were found in 13 (11%) patients, and 10 of the 13 patients exhibited ERBB2 gene amplification. Eight patients with ERBB2 amplification received anti-HER2 therapy combined with basic chemotherapy, and the application of trastuzumab achieved a disease control rate of 7/8. Specifically, the stable disease rate was 1/8, the partial response rate was 6/8, and the complete response rate was 0/8. One patient exhibited grade 3 neutropenia, and two patients with partial responses underwent exploratory laparotomy after systemic treatment. The tumor from one patient remained unresectable after surgical exploration, and the other patient accepted radical hemihepatectomy treatment. The patient had not experienced recurrence as of the analysis date. The median PFS time for trastuzumab-treated patients was 7.3 (1–27) months.

BRCA1/2 mutations were detected in 16 (13%) patients, and most of these mutations were somatic (14 patients, 11%). One patient with a BRCA2 germline mutation had previously underwent mastectomy for breast cancer before the diagnosis of ICC. Four patients accepted poly ADP-ribose polymerase (PARP) inhibitor (olaparib) treatment combined with chemotherapy. Two patients achieved stable disease, and two patients responded partially. Herein, no cases of complete response or disease progression and no grade 3–4 toxic effects were observed.

KRAS/NRAS/BRAF alterations occurred in 36 (30%) patients. Among the 86 patients with no mutations, 22 accepted cetuximab treatment combined with chemotherapy. The disease control and response rates were 78% and 47%, respectively, which were higher than those achieved with chemotherapy alone (72% and 11%); the differences were not statistically significant (P = 0.16), and no complete response was observed. Patients in the cetuximab group had a longer PFS time than those in the chemotherapy group (9.0 vs 6.7 months), but the difference was not statistically significant (P = 0.51, Additional file 4: Figure S2). Grade 3 toxic effects included rashes (n = 5) and neutropenia (n = 2), and no grade 4 toxic effects were observed.

One patient acquired an NRAS mutation in the 5th month of cetuximab treatment, which was diagnosed via imaging and further confirmed by NGS (Additional file 5: Figure S3).

**Fig. 4:**
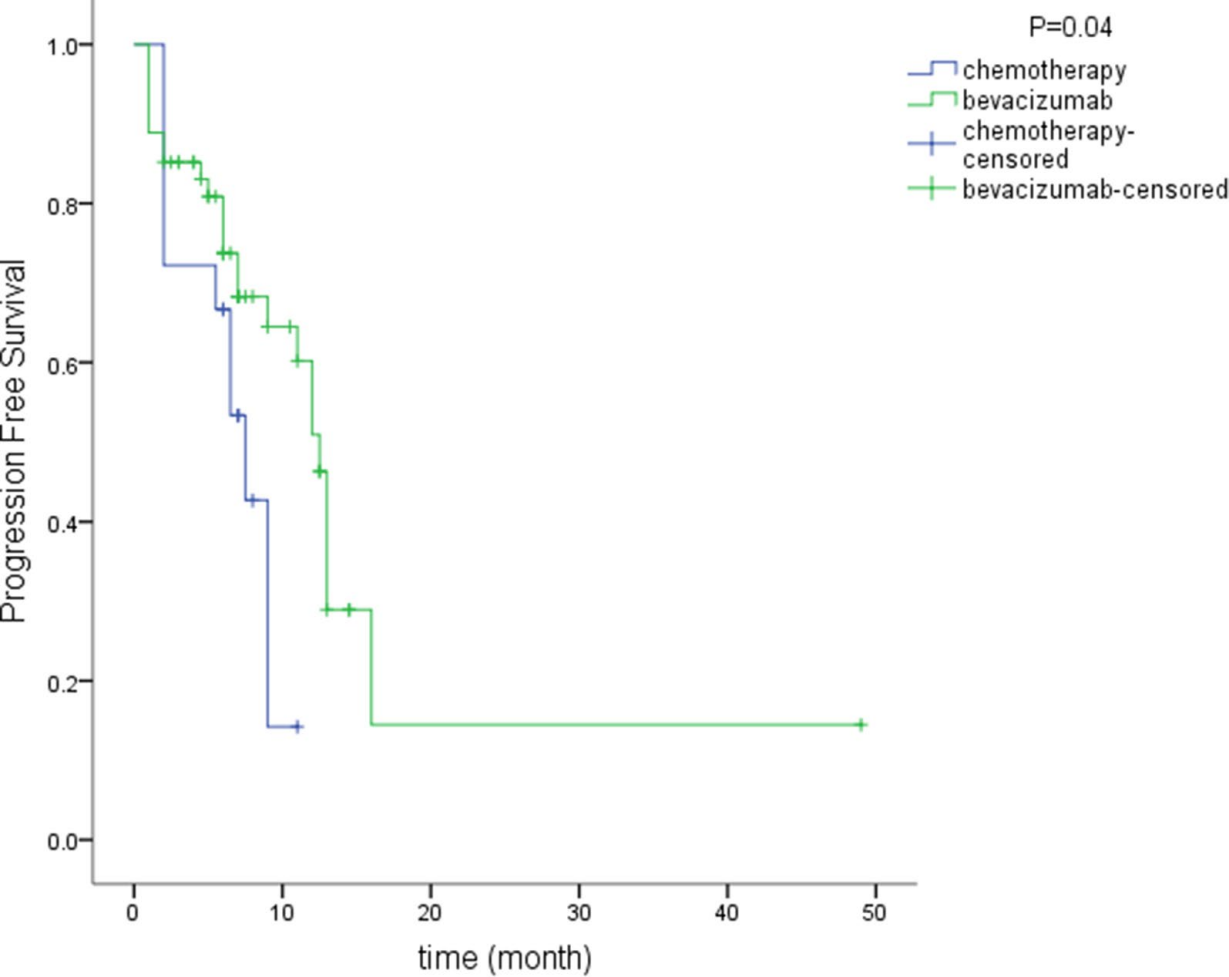
PFS was 15.4 months in the bevacizumab group and 6.7 months in the chemotherapy group (P = 0.04)

**Fig. 5:**
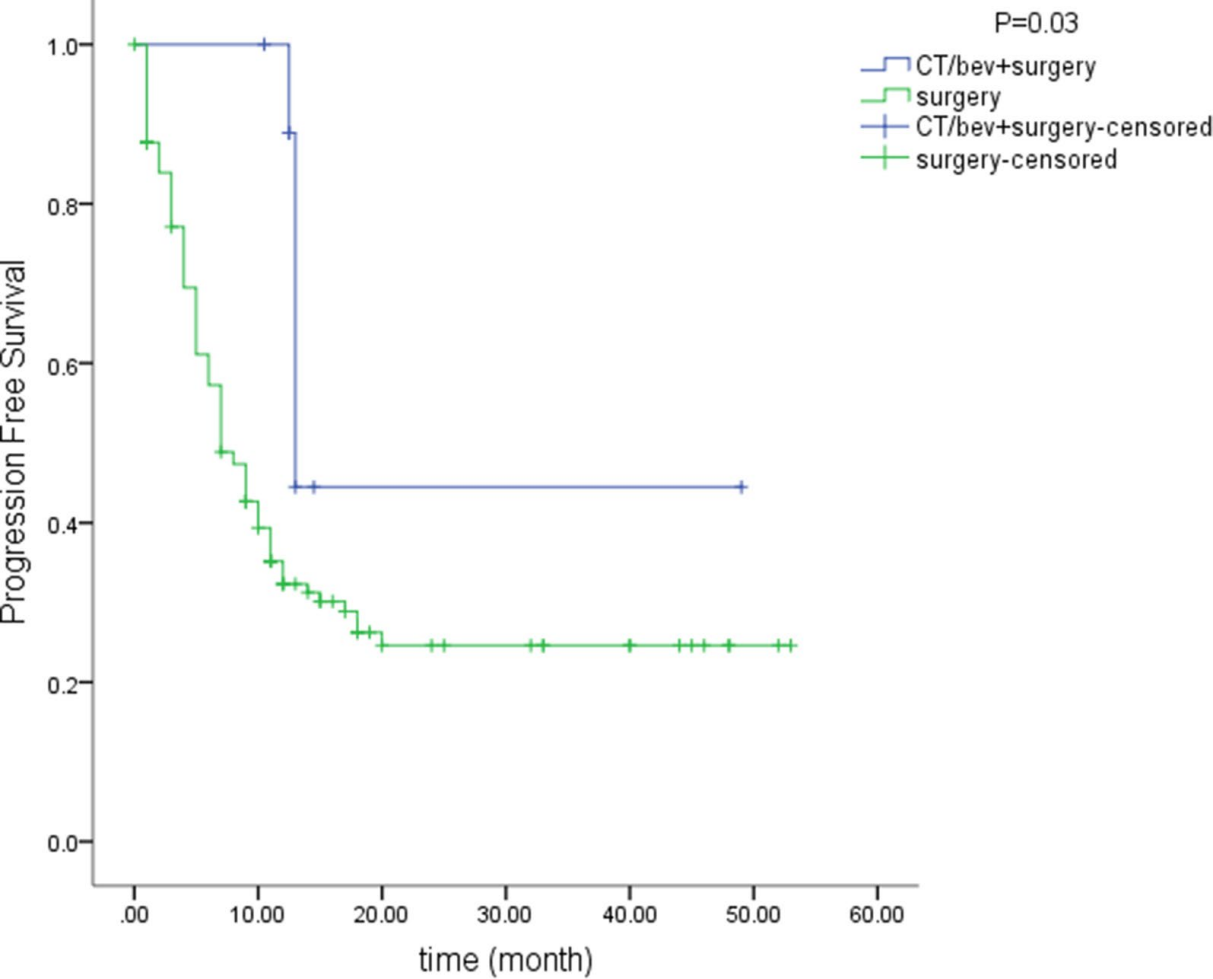
Patients who underwent preoperative systemic treatment had a longer PFS than those who underwent surgical treatment alone (28.9 vs 18.0 months, P = 0.03)

Fifty-four patients received anti-VEGF treatment (bevacizumab) combined with chemotherapy, and the disease control and response rates were 85% and 60%, respectively. KRAS/NRAS/BRAF were wild-type in 33/54 patients and mutated in 21/54 patients. Two (4%) patients had complete responses, and 30 (56%) patients had partial responses. At 6 weeks after treatment, one patient had a stroke, which was diagnosed as a grade 3 adverse event; VEGFA amplification was detected in this patient’s specimen. After 4 weeks of treatment, one patient exhibited acute kidney failure, which was diagnosed as a grade 5 adverse event. The clinical features of the patient indicated tumor lysis syndrome (Additional file 6: Figure S4). Other grade 3 toxic effects included neutropenia (n = 2) and hypertension (n = 1). Patients in the bevacizumab group had a better therapeutic effect (P = 0.001) and a longer PFS time (15.4 vs 6.7 months, P = 0.04) than those in the chemotherapy group (Fig. 4). After systemic treatment, nine (17%) patients with partial responses and one (2%) patient with a complete response underwent exploratory laparotomy followed by hepatectomy. Pathological diagnosis revealed tumor activity in the patient with a clinically complete response. We compared the PFS time of the 10 patients with 139 patients accepted radical hepatectomy treatment in our center. Patients accepted pre-operation systemic treatment acquired a longer PFS time compare to patients accepted surgery treatments along (28.9 vs 18.0 months, P = 0.03; Fig. 5). The therapeutic regimens used based on genetic alterations were summarized in Table 3.

**Table 3:** Therapeutic regimens used based on genetic alterations

Therapeutic regimen	Genetic alterations	Patient number
Cetuximab	KRAS/NRAS/BRAF wild type	22
Trastuzumab	ERBB2 amplification	8
Olaparib	BRCA1/2 mutation	4
Pembrolizumab	MSI-H	2

**Fig. 6:**
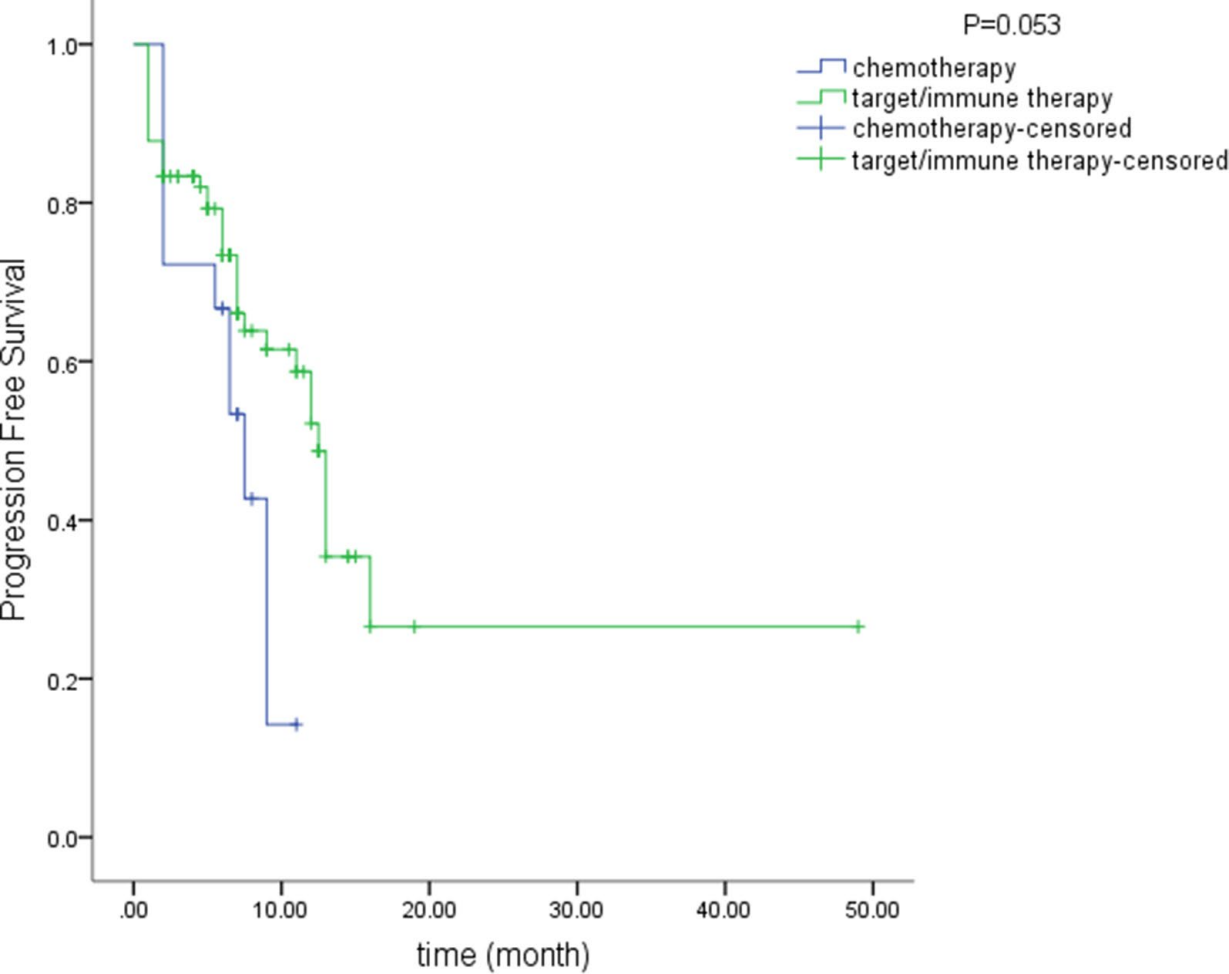
Overall, patients who received targeted or immunotherapy agents had a longer PFS than those who received chemotherapy (19.3 vs 6.7 months, P = 0.053)

Furthermore, overall patients accepted target or immune therapy agencies (n = 90) had a longer PFS than those in the chemotherapy group (n = 18; 19.3 vs 6.7 months). There was an obvious tendency, but the difference was not statistically significant (P = 0.053, Fig. 6).

## Discussion

HBV, hepatitis C virus (HCV) and chronic cholangitis contribute to the majority of the 70,000 ICC cases emerging in China [1], revealing the heterogeneity of Chinese cases with those of Westerners and South East Asians. Multiple studies have investigated the molecular profiles of cholangiocarcinoma in Western countries, but genetic information for Chinese patients with ICC is lacking. In this study, 417 cancer-associated genes from 122 ICC patient samples were subjected to NGS, and a genetic profile was obtained. TP53, KRAS and ARID1A were identified as commonly occurring genetic alterations, similar to the results of the present study (Table 4).

**Table 4:** Most commonly altered genes in intrahepatic cholangiocarcinoma

	Lowery et al. [14] (N = 158)	Churi et al. [37] (N = 55)	Ross et al. [38] (N = 28)	Zou et al. [39] (N = 102)
TP53 (34%), (%)	20	29	36	38
KRAS (25%), (%)	7	24	11	17
ARID1A (17%), (%)	23	20	36	7

Genomic alterations, mostly rearrangements, in FGFR2 are commonly reported in cholangiocarcinoma patients in Western countries [14, 15], and these alterations result in constitutive activation of the FGFR2 receptor. In this study, a much lower FGFR2 alteration rate (2%) was detected in the Chinese population compared with that in other populations. Patients with FGFR fusion could benefit from BGJ398 and derazantinib treatment, and prospective clinical trials are evaluating the efficacy of multiple anti-FGFR treatments on cholangiocarcinoma (NCT04093362/NCT02924376/NCT03773302/NCT04238715/NCT02150967/NCT03230318/NCT03656536/NCT04088188).

ERBB2 amplifications are relatively rare in ICC compared with other types of biliary tract cancer (BTC) [16]. Due to low mortality and the number of patients with specific ERBB2 amplifications, a previous clinical trial (NCT00478140) was halted. The frequency of ERBB2 amplification in this study was 8%, which is higher than that previously reported by other studies^16^. Notably, the disease control rate among eight patients who received anti-ERBB2 treatment with trastuzumab reached 7/8, and one patient achieved a clinically resectable status. The therapeutic efficacy of anti-ERBB2 agents on ERBB2-amplified ICCs has not been previously reported, but phase II trials in China and South Korea are set to evaluate the combination of gemcitabine-based chemotherapy with trastuzumab in cases of ERBB2-amplified extrahepatic cholangiocarcinoma and gallbladder cancer (NCT02836847) and BTC (NCT03613168). Basket and multicenter trials may evaluate this treatment more adequately.

Cholangiocarcinoma patients with BRCA1/2 mutations treated with PARP inhibitors exhibited a favorable response [17]. The predictive features of germline and somatic mutations remain to be elucidated, and the germline versus somatic mutation ratio in this study was 2:12. In this study, only four patients accepted olaparib treatment, mostly due to economic factors. A recent study demonstrated that the accumulation of 2-hydroxyglutarate in association with isocitrate dehydrogenase (IDH) mutations can suppress homologous recombination and thereby induce sensitivity to PARP inhibitors [18]. These findings provided the foundation for a trial exploring the antitumoral activity of olaparib in solid tumors that harbor IDH1/2 mutations (NCT03212274). IDH1/2 alterations are frequently reported in ICC, and 13 (11%) patients with IDH1 mutations and 6 (5%) patients with IDH2 mutations were included in this study. Ongoing studies are evaluating the IDH1 inhibitors ivosidenib (NCT02989857) and BAY 1436032 (NCT02746081) in BTC. However, because these IDH inhibiters are not available at our center, our patients did not have the opportunity to receive anti-IDH treatments.

In this study, compared with those in the chemotherapy alone group, the partial response and stable disease rates were higher and the PFS time was longer in cetuximab treated patients; however, the differences were not statistically significant. These results were obtained from patients with wild-type KRAS/NRAS/BRAF genes. Both NRAS and BRAF were altered in 3 (2%) patients, and this rate was much lower than that of KRAS alterations (31 patients, 25%). Previous clinical trials indicated that EGFR inhibitors, including cetuximab and erlotinib, do not improve the therapeutic efficacy of gemcitabine-based chemotherapy, although they are well tolerated [19, 20]. One study indicated that the KRAS status was not associated with PFS in cholangiocarcinoma patients treated with both gemcitabine and cetuximab, but the patient sample number was limited [21]. A phase II study reported that panitumumab combined with gemcitabine and oxaliplatin (GEMOX) had a good therapeutic efficacy in KRAS wild-type patients, who exhibited a good survival time [22]. Our results support further investigations of the administration of EGFR inhibiters to KRAS wild-type patients.

The disease control rate (85%) and response rate (60%) achieved with bevacizumab treatment combined with chemotherapy in this study as well as the prolonged PFS time (15.4 months) are encouraging. The survival data of patients treated with this combination were significantly better than those of patients in the chemotherapy group. Moreover, two of the patients exhibited complete responses, and 10 patients underwent hepatectomy after systemic therapy. However, the evidence for adding bevacizumab to gemcitabine-based first-line chemotherapy is still insufficient and comparatively weak. Zhu et al. assessed the efficacy of bevacizumab in combination with GEMOX in 35 patients with advanced BTCs, and the reported PFS rate at 6 months was 63%, which was satisfactory but below the targeted rate of 70% [23]. Iyer et al. explored the efficacy of bevacizumab combined with gemcitabine-capecitabine in 50 patients with advanced BTCs, reporting a PFS time of 8.1 months, an OS time of 10.2 months and a clinical benefit rate of 72% [24]. However, concluding that the addition of bevacizumab improved outcomes was not possible based on the results. A controlled clinical trial reported longer PFS times in the bevacizumab combination group (6.48 months) than in the GEMOX group (3.72 months) [25]. There are cases of ICCs being successfully treated with bevacizumab combined with oxaliplatin-based chemotherapies as a consequence of misdiagnosed colorectal carcinoma liver metastasis [26, 27]. Evidence supporting neoadjuvant or conversional treatment in ICC is lacking, but our results showed that late staged patients accepted combination of chemotherapy and bevacizumab treatment and consecutive operation had a longer PFS time than those underwent radical surgery. Patients may benefit from pre-operation systemic treatment. Thus, bevacizumab is an underutilized target agent for ICCs, and its efficacy and safety should be further evaluated.

The reported frequency of MSI-H in cholangiocarcinoma patients ranges widely from 1% to 10% [28, 29], and a prospective study reported an MSI-H rate of 0.5% in unselected cholangiocarcinoma patients [14]. In this study, the MSI-H rate was 2% in ICC patients; both patients accepted immunotherapy with pembrolizumab, and the therapeutic efficacy was satisfactory. The FDA approved pembrolizumab for the treatment of MSI-H/mismatch repair deficient (dMMR) tumors, which showed a predictive response to immunotherapy with PD-1 checkpoint inhibitors [30]. The KEYNOTE16 phase II study reported a 100% disease control rate for 4 MSI-H cholangiocarcinoma patients who received pembrolizumab [6], and ongoing clinical trials are accessing immunotherapy in combination with chemotherapy (NCT03111732) or radiotherapy (NCT03898895) for the treatment of cholangiocarcinoma. Additionally, a trial has been designed that combines a PD-1 antibody, a tyrosine kinase inhibitor (TKI, lenvatinib) and GEMOX for the treatment of ICCs (NCT0395197). All studies accessing the therapeutic efficacies of TKIs (including the multitarget TKIs cabozantinib [31], vandetanib [32], sorafenib [33]; the panErbB family receptor TKI afatinib [34]; the VEGF family receptor TKI cediranib [35]; and the combination of pazopanib and trametinib [36]) failed to show survival improvements. The use of checkpoint inhibitor and TKIs in combination may bring hope to patients with ICC.

This study was retrospective, which limits its evidence grade. However, we herein integrated the genetic profiles of ICC patients in the Chinese population. To our knowledge, this is the largest study on the use of NGS results as a reference for selecting target/immune therapies.

## Conclusions

The therapeutic efficacies of personalized treatments are encouraging. Notably, anti-VEGF therapy showed promising improvements in regards to tumor response and patient survival rates. In summary, patients can benefit from NGS customized therapy, and further investigations are crucial for future strategies.

## Supplementary information

**Additional file 1: Table S1.** Gene list of the 417-gene panel.

**Additional file 2: Table S2.** Therapeutic strategies.

**Additional file 3: Figure S1.** Portal vein tumor thrombosis (red arrows) and tumor (green arrow) shrinkage during treatment.

**Additional file 4: Figure S2.** Progression-free survival was 9.0 months in the cetuximab group and 6.7 months in the chemotherapy group (P = 0.04).

**Additional file 5: Figure S3.** In the first 5 months of cetuximab treatment, tumor size decreased (A and B). After acquiring an NRAS mutation, the patient’s tumor size increased (C).

**Additional file 6: Figure S4.** Tumor shrinkage after 4 weeks (2 cycles) of treatment of GEMOX and bevacizumab (red arrow). Green arrow points the gallbladder.

## Data Availability

The datasets used and analysed during the current study are available from the corresponding author on reasonable request.

## Abbreviations

BTC: Biliary tract cancer
CTCAE: Common Terminology Criteria for Adverse Events
dMMR: MSI-H/mismatch repair deficient
FDR: False discovery rate
FFPE: Formalin-fixed, paraffin-embedded
FUSCC: Fudan University Shanghai Cancer Center
gDNA: Genomic DNA
GEMOX: Gemcitabine and oxaliplatin
HBV: Hepatitis B virus
HCV: Hepatitis C virus
HGVS: Human Genome Variation Society
ICC: Intrahepatic cholangiocarcinoma
ICs: Immune cells
IDH: Isocitrate dehydrogenase
MSI: Microsatellite instability
MSS: Microsatellite stable
NCCR: National Central Cancer Registry of China
NGS: Next-generation sequencing
OS: Overall survival
PARP: Poly ADP-ribose polymerase
PCR: Polymerase chain reaction
PD-L1: Programmed death ligand 1
PFS: Progression-free survival
RECIST: Response Evaluation Criteria in Solid Tumors
SNVs: Single-nucleotide variation
TCs: tumor cells
TKI: Tyrosine kinase inhibitor
TMB: Tumor mutational burden
VAF: Variant allele frequency
