# Supplementary material for: Comprehensive molecular profiling of intrahepatic cholangiocarcinoma in the Chinese population and therapeutic experience
Source: J Transl Med. 2020 Jul 6;18:273. doi: 10.1186/s12967-020-02437-2 (PMC7336472; doi:10.1186/s12967-020-02437-2)
Supplement: Supplementary file 1 — Additional file 1: Table S1. Gene list of the 417-gene panel. [file 12967_2020_2437_MOESM1_ESM.docx]

Additional file 1: Table S1. Gene list of the 417-gene panel

|  | | | | | | | | | | | | | |
| --- | --- | --- | --- | --- | --- | --- | --- | --- | --- | --- | --- | --- | --- |
| ALK | ATM | ATR | BRCA1 | BRCA2 | BRIP1 | CD274 | CHEK2 | DNMT3A | EGFR | FANCA | JAK1 | JAK2 | KRAS |
| MDM2 | MDM4 | MLH1 | MSH2 | MSH6 | PALB2 | PDCD1LG2 | PMS2 | POLD1 | POLE | PTEN | RAD50 | TP53 |  |
|  | | | | | | | | | | | | | |
| ABL1 | AKT1 | AKT2 | ALK | AR | ARAF | ATM | ATR | BCL2L11 | BRAF | BRCA1 | BRCA2 | BRIP1 | CCND1 |
| CD274 | CDK12 | CDK4 | CDK6 | CDKN1B | CDKN2A | CHEK1 | CHEK2 | DDR2 | DNMT3A | EGFR | EPHA2 | ERBB2 | ERBB3 |
| ERBB4 | ERRFI1 | ESR1 | FBXW7 | FGFR1 | FGFR2 | FGFR3 | FLT3 | GLI1 | GLI2 | GLI3 | HRAS | IDH2 | JAK1 |
| JAK2 | KDR | KIT | KRAS | MAP2K1 | MAP2K2 | MCL1 | MDM2 | MDM4 | MET | MLH1 | MRE11A | MSH2 | MSH6 |
| MTOR | NF1 | NF2 | NFKBIA | NKX2-1 | NRAS | NTRK1 | NTRK2 | NTRK3 | PALB2 | PDCD1LG2 | PDGFRA | PDGFRB | PIK3CA |
| PIK3R1 | PIK3R2 | PMS2 | POLD1 | POLE | PTCH1 | PTEN | RAD50 | RARA | RET | ROS1 | RPTOR | SMO | SRC |
| STK11 | TP53 | TSC1 | TSC2 | VEGFA | VHL |  |  |  |  |  |  |  |  |
|  | | | | | | | | | | | | | |
| ARID1A | ATM | ATR | BRCA1 | BRCA2 | CHEK1 | CHEK2 | CYP2C19 | CYP2D6 | DPYD | FANCD2 | FANCF | MLH1 | MRE11A |
| PRKDC | RAD50 | TOP2A | TPMT | UGT1A1 | WEE1 | ZNF217 |  |  |  |  |  |  |  |
|  | | | | | | | | | | | | | |
| ATM | ATR | AURKA | BAP1 | BARD1 | BLM | BRCA1 | BRCA2 | BRIP1 | CDK12 | CHEK1 | CHEK2 | CUL3 | ERCC1 |
| FANCA | FANCC | FANCD2 | FANCE | FANCF | FANCG | FANCL | MLH1 | MRE11A | MSH2 | MSH6 | MUTYH | PALB2 | PMS2 |
| POLD1 | POLE | PRKDC | RAD50 | RAD51 |  |  |  |  |  |  |  |  |  |
|  | | | | | | | | | | | | | |
| ABL2 | ACVR1B | ACVR2A | ADAM29 | ADGRA2 | AKT3 | AMER1 | APC | ARFRP1 | ARID1B | ARID2 | ASXL1 | ATRX | AURKB |
| AXIN1 | AXL | BCL2 | BCL2L1 | BCL2L2 | BCL6 | BCOR | BCORL1 | BCR | BIRC5 | BLK | BMX | BRD4 | BTG1 |
| BTK | CARD11 | CBFB | CBL | CCND2 | CCND3 | CCNE1 | CD79A | CD79B | CDC73 | CDH1 | CDK8 | CDKN1A | CDKN2B |
| CDKN2C | CEBPA | CHD2 | CHD4 | CIC | CRBN | CREBBP | CRKL | CRLF2 | CSF1R | CSK | CSNK1A1 | CTCF | CTNNA1 |
| CTNNB1 | CXCR4 | CYLD | DAXX | DDR1 | DICER1 | DOT1L | EGF | EMSY | EP300 | EPHA3 | EPHA5 | EPHA7 | EPHB1 |
| ERG | ETV1 | ETV4 | ETV5 | ETV6 | EZH2 | FAM135B | FAM46C | FAS | FAT1 | FGF10 | FGF14 | FGF19 | FGF23 |
| FGF3 | FGF4 | FGF6 | FGFR4 | FGR | FH | FLCN | FLT1 | FLT4 | FOXL2 | FOXP1 | FRS2 | FUBP1 | FYN |
| GABRA6 | GATA1 | GATA2 | GATA3 | GATA4 | GATA6 | GID4 | GNA11 | GNA13 | GNAQ | GNAS | GRIN2A | GRM3 | GSK3B |
| H3F3A | HCK | HGF | HNF1A | HSD3B1 | HSP90AA1 | IDH1 | IGF1R | IGF2 | IKBKE | IKZF1 | IL7R | INHBA | INPP4B |
| IRF2 | IRF4 | IRS2 | ITK | JAK3 | JUN | KAT6A | KDM5A | KDM5C | KDM6A | KEAP1 | KEL | KLHL6 | KMT2A |
| KMT2C | KMT2D | LCK | LIMK1 | LMO1 | LRP1 | LRP1B | LYN | LZTR1 | MAGI2 | MAP2K4 | MAP3K1 | MAP4K5 | MED12 |
| MEF2B | MEN1 | MITF | MPL | MS4A1 | MST1R | MYB | MYC | MYCL | MYCN | MYD88 | NEK11 | NFE2L2 | NOTCH1 |
| NOTCH2 | NOTCH3 | NPM1 | NRG1 | NRG3 | NSD1 | NUP93 | PAK3 | PARK2 | PAX5 | PBRM1 | PDK1 | PIK3C2B | PIK3CB |
| PIK3CD | PIK3CG | PKD2 | PLA2G1B | PLCG2 | PPP2R1A | PRDM1 | PREX2 | PRKAR1A | PRKCI | PRSS8 | PTK2 | PTK6 | PTPN11 |
| QKI | RAC1 | RAF1 | RANBP2 | RB1 | RBM10 | RICTOR | RIT1 | RNF43 | ROCK1 | ROCK2 | RUNX1 | RUNX1T1 | RXRA |
| SDHA | SDHB | SDHC | SDHD | SETD2 | SF3B1 | SIK1 | SLIT2 | SMAD2 | SMAD3 | SMAD4 | SMARCA2 | SMARCA4 | SMARCB1 |
| SNCAIP | SOCS1 | SOX10 | SOX2 | SOX9 | SPEN | SPOP | SPTA1 | SRMS | STAG2 | STAT3 | STAT4 | STK24 | SUFU |
| SYK | TAF1 | TBX3 | TCF7L2 | TEK | TERT | TET2 | TGFBR1 | TGFBR2 | TIE1 | TMPRSS2 | TNFAIP3 | TNFRSF14 | TNFSF11 |
| TNK2 | TOP1 | TSHR | TYK2 | U2AF1 | WEE2 | WISP3 | WT1 | XIAP | XPO1 | YES1 | ZBTB2 | ZNF703 | ZNF750 |
