# Supplementary material for: Comprehensive molecular profiling of intrahepatic cholangiocarcinoma in the Chinese population and therapeutic experience
Source: J Transl Med. 2020 Jul 6;18:273. doi: 10.1186/s12967-020-02437-2 (PMC7336472; doi:10.1186/s12967-020-02437-2)
Supplement: Supplementary file 2 — Additional file 2: Table S2. Therapeutic strategies. [file 12967_2020_2437_MOESM2_ESM.docx]

Additional file 6: Table S2. Therapeutic strategies

|  | Dose | Usage |
| --- | --- | --- |
| Gemcitabine | 1000 mg/m^2^ | d1,8/q3w |
| Cisplatin | 80 mg/m^2^ | d1-3/q3w |
| Oxaliplatin | 100 mg/m^2^ | d1/q3w |
| Capecitabine | 1000 mg/m^2^ bid | d1-14/q3w |
| Pembrolizumab | 200 mg | d1/q3w |
| Trastuzumab | 6 mg/kg | d1/q3w |
| Olaparib | 300 mg bid | qd |
| Cetuximab | 500 mg/m^2^ | d1/q2w |
| Bevacizumab | 10 mg/kg | d1/q3w |
