# Supplementary figures and images for: Comprehensive molecular profiling of intrahepatic cholangiocarcinoma in the Chinese population and therapeutic experience
Source: J Transl Med. 2020 Jul 6;18:273. doi: 10.1186/s12967-020-02437-2 (PMC7336472; doi:10.1186/s12967-020-02437-2)

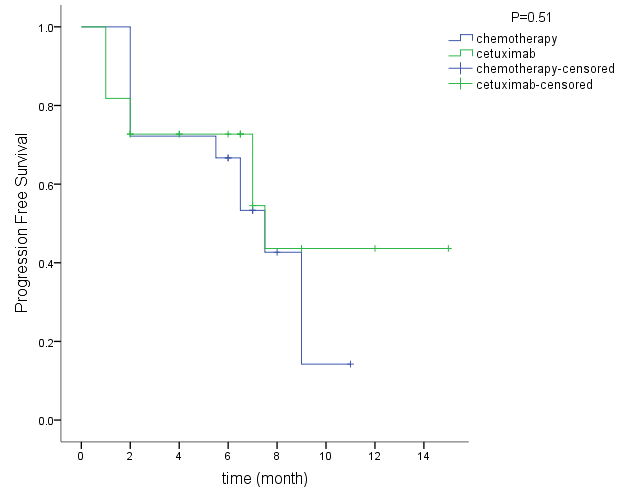

Supplement: Supplementary file 4 — Additional file 4: Figure S2. Progression-free survival was 9.0 months in the cetuximab group and 6.7 months in the chemotherapy group (P = 0.04). [file 12967_2020_2437_MOESM4_ESM.png]
